# Bisphenol A Induces DNA Damage and Differential Cytotoxicity in Thyroid Cells: High‐Dose Effects Challenge Regulatory Thresholds

**DOI:** 10.1002/jat.70163

**Published:** 2026-03-12

**Authors:** Izabela Fernanda Dal' Bó, Natássia Elena Bufalo, Valdemar Máximo, Laura Sterian Ward

**Affiliations:** ^1^ Department of Medicine School of Medical Sciences‐State University of Campinas Campinas São Paulo Brazil; ^2^ Department of Medicine São Leopoldo Mandic and Research Center Campinas São Paulo Brazil; ^3^ Department of Medicine Max Planck University Center Indaiatuba São Paulo Brazil; ^4^ Institute for Research and Innovation in Health (i3S) University of Porto Porto Portugal; ^5^ Institute of Molecular Pathology and Immunology of the University of Porto (Ipatimup) Porto Portugal; ^6^ Department of Pathology Faculty of Medicine of the University of Porto (FMUP) Porto Portugal

**Keywords:** bisphenol A, cytotoxicity, genotoxicity, thyroid cancer, thyroid cells

## Abstract

Bisphenol A (BPA), a ubiquitous environmental endocrine disruptor, has been implicated in carcinogenesis; however, its effects on thyroid cells remain unclear. This study investigated BPA‐induced cytotoxicity and genotoxicity in thyroid cell lines, including normal follicular (Nthy‐ori 3‐1), papillary carcinoma (TPC‐1 and BCPAP) and anaplastic carcinoma (8505C) cells, including exposure to the specific migration limit (SML) established by Brazilian regulations (1 μg/mL). Cells were exposed for 24 and 48 h, and membrane integrity, metabolic activity, and DNA damage were assessed using trypan blue exclusion, CCK‐8, and comet assays, respectively. Nthy‐ori 3‐1 exhibited non‐monotonic cytotoxicity, with 60% mortality at the SML (24 h). The 8505C line displayed notable resistance to membrane damage at high concentrations but was highly sensitive to genotoxicity, which intensified after BPA exposure and may be related to TP53 mutations. The SML dose caused significant DNA damage in normal cells, whereas papillary lines were less affected. These findings demonstrate cell‐context‐dependent BPA effects and suggest that current regulatory thresholds may not adequately protect thyroid health, particularly in individuals with pre‐existing thyroid conditions. BPA's impact involves both metabolic disruption and impaired DNA repair pathways, supporting its potential carcinogenicity.

## Abstract 2

This study evaluates the cytotoxic and genotoxic effects of Bisphenol A (BPA) on thyroid cell lines: normal (Nthy‐ori 3‐1) and carcinoma‐derived (TPC‐1, BCPAP, 8505C)—after 24‐ and 48‐h exposure at concentrations including the Brazilian SML (1 μg/mL). Results reveal cell‐type‐specific responses: SML‐level BPA induced ~60% mortality in normal cells and significant DNA damage across cell types, especially in the TP53‐mutated anaplastic line. The data suggest current regulatory thresholds may inadequately protect thyroid cells, underscoring BPA's potential carcinogenic risk.

## 1 Introduction

Bisphenol A (BPA), a plasticizer widely used in polycarbonates and epoxy resins for products such as plastic bottles, food containers, and thermal receipts, is a pervasive environmental pollutant (Fiore et al. 2019; Vandenberg et al. 2007). Ubiquitous human exposure occurs through multiple routes, with fetuses and children facing heightened risks owing to elevated levels of unconjugated BPA (Vandenberg et al. 2012). As an endocrine disruptor, BPA mimics endogenous hormones, disrupts their synthesis and secretion, and interferes with endocrine system function (Kim and Park 2019). Specifically, BPA antagonizes the thyroid hormone signaling pathways (Moriyama et al. 2002). It directly affects thyrocytes by upregulating genes critical for thyroid hormone biosynthesis such as *sodium iodide symporter* (*NIS*), *thyroglobulin* (*Tg*), and *thyroperoxidase* (*TPO*), as well as transcription factors such as *Pax8*, *Nkx2‐1*, and *Foxe1*, which regulate thyroid development and follicular cell differentiation (Gentilcore et al. 2013; Porreca et al. 2016). Emerging evidence also highlights the genomic and non‐genomic mechanisms at low doses, including micronucleus formation and mitotic abnormalities in endothelial cells (Ribeiro‐Varandas et al. 2013; Sheng et al. 2019).

Epidemiological and experimental studies have suggested a potential link between BPA exposure and thyroid cancer risk; however, these findings remain inconsistent (Kim and Park 2019; Marotta et al. 2019). While Chinese cohort studies have reported elevated urinary BPA levels in patients with thyroid nodules or cancer compared to controls (Li et al. 2019; Zhou et al. 2017), other studies have failed to replicate these associations (Andrianou et al. 2016). Animal models further illustrate this ambiguity: BPA enhances susceptibility to thyroid tumors (Zhang, Wei, et al. 2017) and promotes thyroid cancer cell proliferation (Zhang, Zhang, et al. 2017); however, it does not directly induce carcinogenesis in DHPN‐stimulated models (Takagi et al. 2002).

Despite emerging evidence, critical gaps persist regarding the molecular mechanisms underlying BPA and its differential effects on normal and mutated thyroid cells. Notably, BPA may exert distinct effects depending on cellular mutation profiles (Li et al. 2021), yet systematic comparisons across cell types and concentrations have been lacking. Given the ubiquity of BPA exposure, it is imperative to understand its concentration‐dependent cytotoxicity and genotoxicity in both normal and neoplastic thyroid cells (Gorini et al. 2020).

This study addresses these gaps by evaluating BPA‐induced DNA damage and functional responses in thyroid cell lines representing normal follicular epithelium and various cancer phenotypes.

## 2 Material and Methods

### 2.1 Cell Culture and Experimental Conditions

Four human thyroid cell lines were used in this study. The normal thyroid follicular epithelial cell line Nthy‐ori 3‐1 (RRID:CVCL_2659), was purchased from Sigma‐Aldrich (St. Louis, MO, USA; Cat# 90011609). The neoplastic cell lines TPC‐1 (papillary thyroid carcinoma harboring a *RET/PTC1* rearrangement) (RRID:CVCL_6298), BCPAP (papillary thyroid carcinoma harboring the BRAF V600E mutation) (RRID:CVCL_0153), and 8505C (an anaplastic thyroid carcinoma cell line) (RRID:CVCL_1054) were generously provided by Prof. Dr. Valdemar de Jesus Conde Máximo (Institute of Molecular Pathology and Immunology, Ipatimup, University of Porto, Portugal). All cell lines were maintained at 37°C in a humidified atmosphere of 5% CO_2_. Cells were cultured in RPMI 1640 Medium, GlutaMAX Supplement, HEPES from Gibco (Paisley, Scotland, UK, Cat# 72400120), supplemented with 10% fetal bovine serum (Sigma‐Aldrich, Cat# F9665), 1% penicillin–streptomycin (Gibco, Cat# 15140122), and 250 μg/mL fungizone (Gibco, Cat# 15290026). Adherent cultures were passaged using 0.05% trypsin–EDTA (Gibco, Cat# 25300062).

Bisphenol A (BPA; ≥ 99% purity, CAS No. 80‐05‐7) was obtained from Sigma‐Aldrich. A stock solution was prepared in dimethyl sulfoxide (DMSO) and subsequently diluted in the culture medium to achieve the desired treatment concentrations. The concentrations tested included the Specific Migration Limit (SML = 1 μg/mL), a regulatory threshold established by the Brazilian National Health Surveillance Agency (ANVISA). This concentration was derived from the legislated Specific Migration Limit of 0.6 mg/kg, converted for in vitro application using a formula based on the average adult body weight (70 kg) and total body water (42 L) (Agência Nacional de Vigilância Sanitária [ANVISA] 2012; Hall and Guyton 2011).

### 2.2 Cell Viability Assay (Trypan Blue Exclusion)

Cell viability was assessed using Trypan blue exclusion. Briefly, cells were seeded into 12‐well plates at a density of 3.7 × 10^4^ cells/well and allowed to adhere for 24 h. Following adhesion, the cells were treated with 14 concentrations of BPA, ranging from 0.4 to 400 μg/mL, for 24 or 48 h. After treatment, the cells were collected, and a 10 μL aliquot of the cell suspension was mixed with an equal volume of 0.4% Trypan blue solution (Sigma‐Aldrich, Cat# T8154). Viable (unstained) and non‐viable (blue‐stained) cells were counted automatically using a Countess II FL Automated Cell Counter from Thermo Fisher Scientific (Bothell, WA, USA). All treatments were performed in triplicates.

### 2.3 Metabolic Activity Assay (CCK‐8)

Cytotoxicity based on cellular metabolic activity was evaluated using a Cell Counting Kit‐8 (CCK‐8, #96992, Sigma‐Aldrich). The assay was performed in accordance with the manufacturer's instructions in technical and biological triplicate. All cell lines were exposed to BPA at concentrations selected from the trypan blue test results at 24 and 48 h. The 10 μL of CCK‐8 solution was added, and the cells were incubated for 3 h in a CO_2_ incubator. A microplate reader (ELx808, Biotek, Winooski, VT, USA) was used to measure optical density (OD) at 450 nm. The cell viability for BPA was represented as % cell viability compared to that of the control cells as follows:

$$\text{Cell viability}\;\left(\%\right)=\frac{\mathrm{OD}\;\left(\mathrm{BPA}\right)-\mathrm{OD}\;\left(\text{Blank}\right)}{\mathrm{OD}\;\left(\text{Control}\right)-\mathrm{OD}\;\left(\text{Blank}\right)}\times 10$$

### 2.4 Genotoxicity Assessment (Comet Assay)

DNA damage was assessed using the Single Cell Gel Electrophoresis (SCGE) assay, adapted from the protocol of (Singh et al. 1988). Cells were seeded in 6‐well plates at a density of 1 × 10^5^ cells per well and incubated for 24 h. Following incubation, the cells were treated with the selected BPA concentrations for 24 or 48 h. A negative control (untreated cells) and a positive control (cells exposed to 100‐μM hydrogen peroxide (H_2_O_2_; Sigma‐Aldrich, CAS: 7722‐84‐1) for 5 min) were included in each experiment.

After treatment, the cells were washed with 1 mL of phosphate‐buffered saline (PBS) and detached using 1 mL of trypsin–EDTA. The cell suspension was transferred to a polypropylene tube, neutralized with RPMI 1640 medium, and centrifuged at 1200 rpm for 5 min. The supernatant was carefully aspirated, leaving the pellet behind on ice until it was mounted.

For slide preparation, 40 μL of the cell suspension was mixed with 140 μL of 0.8% low‐melting point agarose (LMA). A 100‐μL aliquot of this mixture was layered onto a microscope slide pre‐coated with a base layer of 1.5% normal‐melting‐point agarose (NMA). The slides were immersed in a freshly prepared chilled lysis solution (2.5‐M NaCl, 100‐mM EDTA, 10‐mM Tris–HCl, pH 10–10.5, with 16.0 mL DMSO and 1.6‐mL Triton X‐100 at pH 10–10.5) freshly prepared and stored in the dark at 4°C for 24 h.

Following lysis, the slides were placed in chilled alkaline electrophoresis buffer (300 mM NaOH, 1 mM EDTA, pH > 13) for 20 min at 4°C to allow DNA unwinding. Electrophoresis was then conducted in the same buffer at 25 V and 300 mA for 20 min. The voltage and current were monitored throughout the run to maintain stable operating conditions. The slides were subsequently neutralized with 0.4 M Tris–HCl (pH 7.5) for 5 min, fixed in absolute ethanol, and air‐dried.

Prior to analysis, the slides were stained with GelRed 10,000X in water (#41003, Biotium, Fremont, CA, USA). Comets were visualized using an Axio Scope A1 epifluorescence microscope from Carl Zeiss (Jena, Thuringia, Germany) equipped with a 510–560 nm excitation filter and a 590 nm barrier filter, using a 40× objective. For each sample, 100 randomly selected nucleoids were scored visually and assigned to one of five damage classes (0–4), from no discernible damage (0) to maximum damage (4), as described previously (Collins et al. 1997; Salvadori et al. 2021). The DNA damage index (DI) was calculated for each experimental condition. The entire assay was performed in duplicate, and all solutions were freshly prepared and used within 15 days.

### 2.5 Statistical Analysis

Statistical analyses were performed using SAS System for Windows version 9.4, SAS Institute Inc., 2002–2012, Cary, NC, USA. The Wilcoxon test for related samples was used to compare the numerical measurements of the concentration and control. To compare the numerical measurements among concentrations, times, and cell lines, an ANOVA test for repeated measures was used. Multiple comparisons were performed using Tukey's test. Frequencies (absolute and percentage) were calculated to compare the concentrations of each cell type and time. The exposure time, concentration, and cell type were evaluated using generalized estimating equations (GEE) for the damage classes. The significance level adopted for all statistical tests was 5%.

## 3 Results

### 3.1 Cytotoxic Effects of BPA on Membrane Integrity

The impact of BPA on cell membrane integrity, assessed via trypan blue exclusion, revealed distinct concentration‐response patterns across the four thyroid cell lines (Figure 1 and Table 1). The neoplastic cell lines (TPC‐1, BCPAP, and 8505C) maintained high viability (> 70%) at concentrations up to 50 μg/mL. In contrast, normal follicular Nthy‐ori 3‐1 cells displayed a pronounced non‐monotonic response, exhibiting significant sensitivity at lower doses. Exposure to the regulatory Specific Migration Limit (SML, 1 μg/mL) resulted in only 37% viability at 24 h. Notably, the anaplastic carcinoma line 8505C demonstrated marked resistance at high concentrations, with over 60% of cells remaining viable following 24‐h exposure to 100–200 μg/mL (Table 1).

**FIGURE 1:**
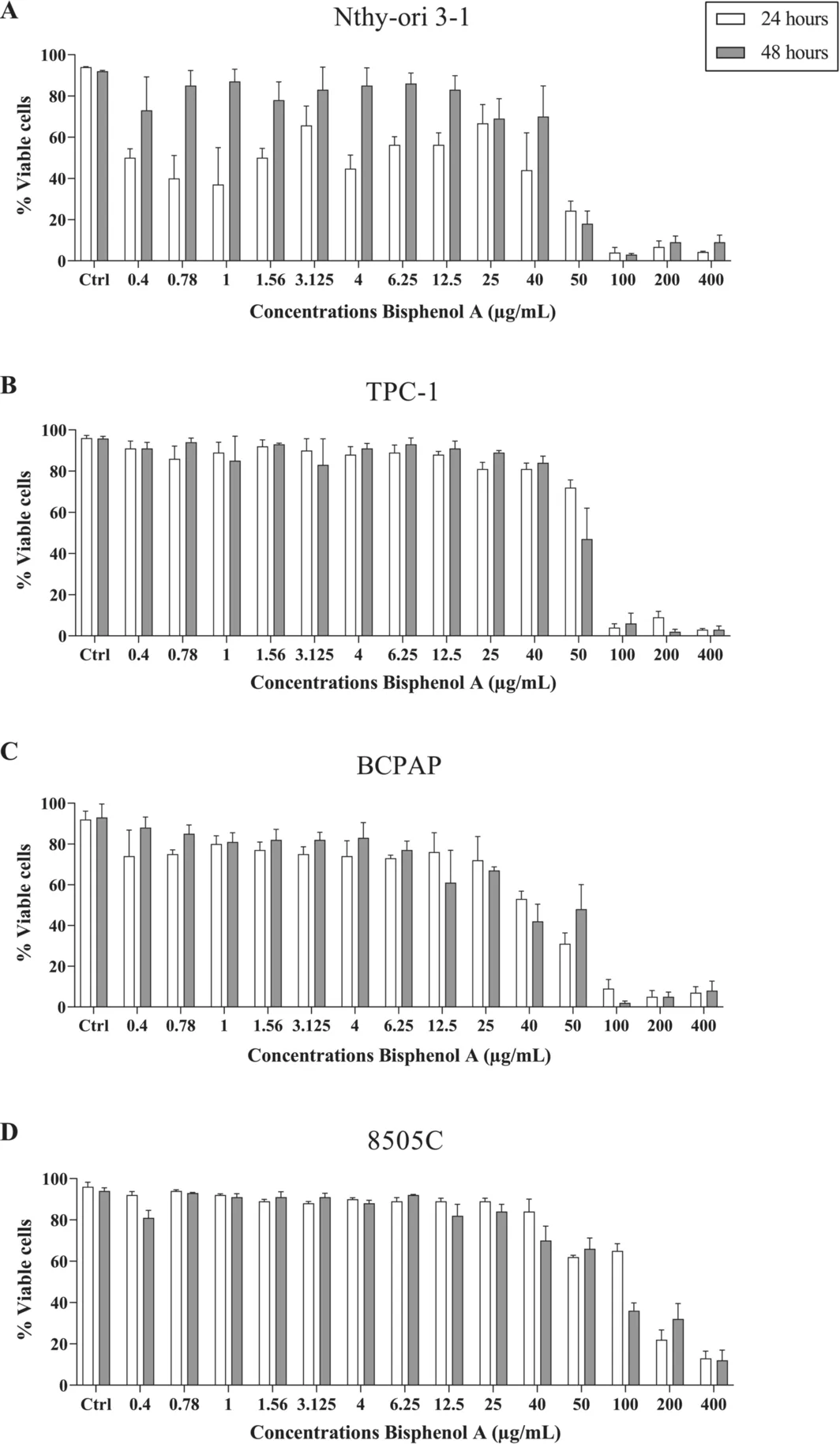
Cell viability of the Nthy‐ori 3‐1 (A), TPC‐1 (B), BCPAP (C), and 8505C (D) cell lines, based on the trypan blue assay, and cell membrane integrity after exposure to BPA for 24 and 48 h. The results are presented as mean ± SE.

**TABLE 1:** Percentage of viable Nthy‐ori 3‐1, TPC‐1, BCPAP, and 8505C cells after exposure to BPA for 24 and 48 h at different concentrations, as measured by Trypan Blue assay.

BPA μg/mL	Nthy‐ori 3‐1		TPC‐1		BCPAP		8505C	
	24 h	48 h	24 h	48 h	24 h	48 h	24 h	48 h
0.4	50%	73%	91%	91%	74%	88%	92%	81%
0.78	40%	85%	86%	94%	75%	85%	94%	93%
1	37%	87%	89%	85%	80%	81%	92%	91%
1.56	50%	78%	92%	93%	77%	82%	89%	91%
3.125	66%	83%	90%	83%	75%	82%	88%	91%
4	45%	85%	88%	91%	74%	83%	90%	88%
6.25	56%	86%	89%	93%	73%	77%	89%	92%
12.5	56%	83%	88%	91%	76%	61%	89%	82%
25	67%	69%	81%	89%	72%	67%	89%	84%
40	44%	70%	81%	84%	53%	42%	84%	70%
50	24%	18%	72%	47%	31%	48%	62%	66%
100	4%	3%	4%	6%	9%	2%	65%	36%
200	7%	9%	9%	2%	5%	5%	22%	32%
400	4%	9%	3%	3%	7%	8%	13%	12%

A striking adaptive response was observed in the Nthy‐ori 3‐1 line. While viability at the SML was 40% at 24 h, it recovered to 87% by 48 h. This pattern of reduced cytotoxicity with prolonged exposure was consistent across all tested concentrations in this cell line, suggesting the activation of compensatory recovery mechanisms.

Statistical analysis integrating all 14 concentrations and both time points (Figure 2) confirmed significant main effects of exposure time, cell type, and BPA concentration (repeated measures ANOVA, *p* < 0.05). Significant interactions were observed between time and concentration, and between concentration and cell type, highlighting the complexity of the response. Post hoc Tukey tests identified specific pairwise differences in the overall sensitivity between the cell lines (Table S1).

**FIGURE 2:**
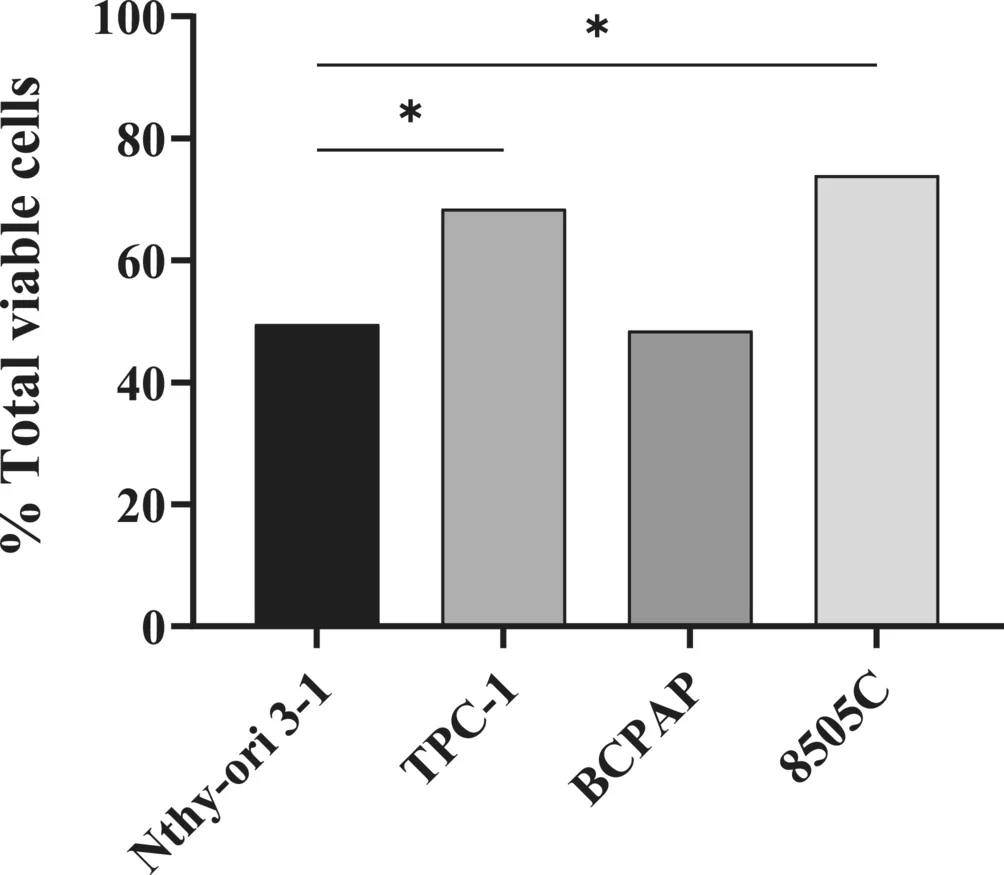
The cellular viability of the four thyroid cell lines exposed to BPA is presented as mean percentages. The comparison included 14 concentrations and two exposure times (24 and 48 h). Repeated measures ANOVA: Results of cell type interactions (Tukey) using the trypan blue assay. * Significant degree of effect for lineage type (Nthy‐ori 3‐1 vs. TPC‐1, *p* = 0.0369; Nthy‐ori 3‐1 vs. 8505C, *p* = 0.0244). No significant difference was observed between (Nthy‐ori 3‐1 vs. BCPAP cells) (*p* = 0.9327). (TPC‐1 vs. BCPAP: *p* = 0.0845); (TPC‐1 vs. 8505C: *p* = 0.9901); and (BCPAP vs. 8505C: *p* = 0.0555).

### 3.2 Influence of BPA on Cellular Morphology

Large groups of thyroid cells observed under an inverted microscope (Axio Scope A1, ZEISS) exhibited rounded morphology. After 24 h of exposure to 1 μg/mL (LME) BPA, neoplastic cells (TPC‐1, BCPAP, and 8505C) appeared similar in confluence and shape to the corresponding non‐exposed control cells (Figure 3A). However, the number of Nthy‐ori 3‐1 cells decreased, with some clustering and displaying a darker color. Morphological changes became more pronounced after treatment with 50 μg/mL of BPA. The compound caused a decrease in the cell number, particularly at concentrations between 40–400 μg/mL, corroborating the results of the cell viability assay. Additionally, cells became rounder, smaller, and lost intercellular connections. Cytoplasmic vacuoles were observed. Generally, after 48 h of exposure, fewer cells were present compared to the 24‐h timepoint, with more cells detaching from the plate. Notably, 8505C cells continued to adhere to the plates (Figure 3B). Treatment starting at 50 μg/mL caused cells to lose their original shape and become increasingly altered, suggesting a dose–response relationship between BPA concentration, cell viability, and thyroid cell line morphology.

**FIGURE 3:**
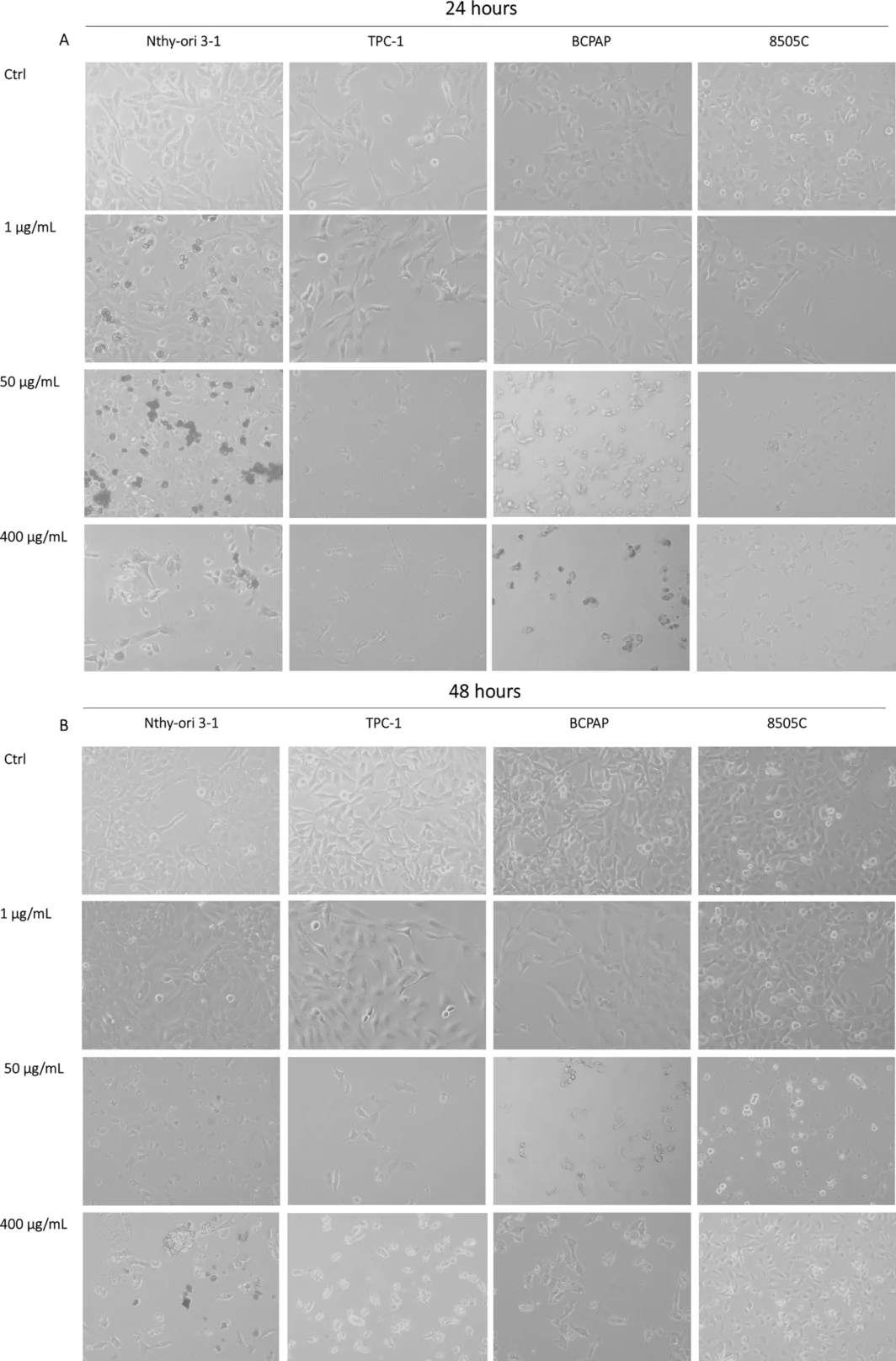
Morphology of thyroid cell lineages. After 24 (A) and 48 h (B) of treatment with different doses of BPA, images were captured using a ZEISS inverted microscope at 40× magnification.

### 3.3 Effects of BPA on Metabolic Activity

Cellular metabolic activity, a key indicator of functional viability, was assessed using the CCK‐8 assay after exposure to a subset of five BPA concentrations (Figure 4 and Table 2). The results revealed a distinct and severe inhibitory effect on the metabolic function of all cell lines.

**FIGURE 4:**
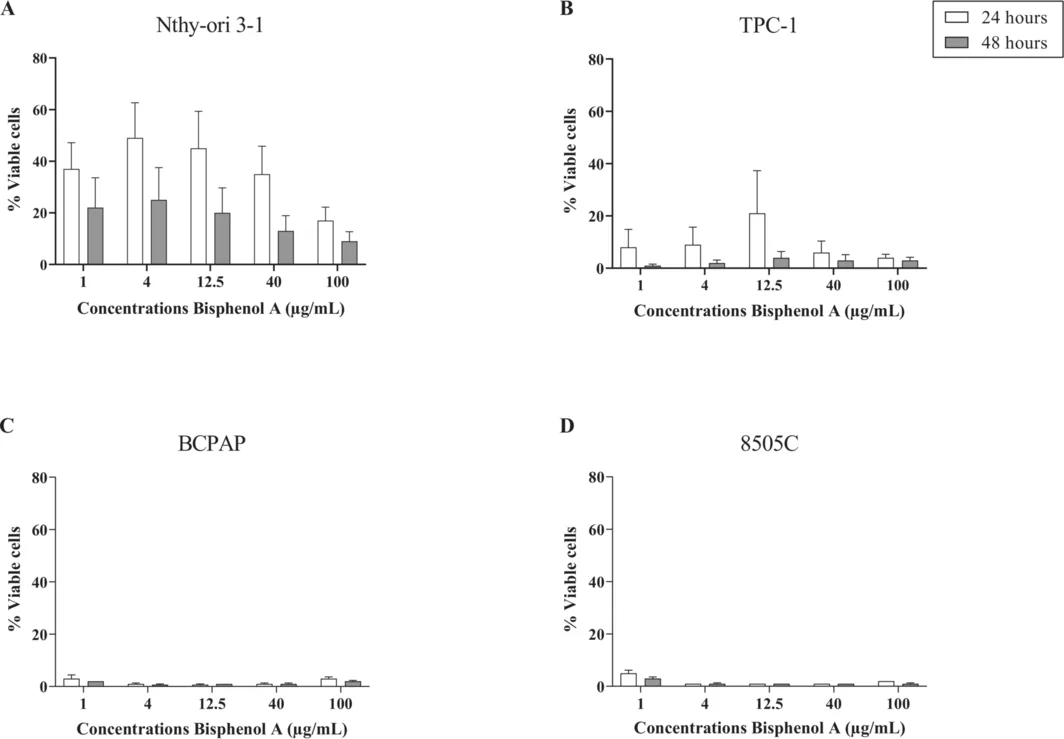
Comparison of the viability of Nthy‐ori 3‐1 (A), TPC‐1 (B), BCPAP (C), and 8505C (D) cells after exposure to increasing concentrations of BPA for 24 and 48 h, as measured by the CCK‐8 assay. The results are presented as mean ± SE.

**TABLE 2:** Percentage of viable cell Nthy‐ori 3‐1, TPC‐1, BCPAP, and 8505C cells after exposure to BPA for 24 and 48 h, as measured by the CCK‐8 assay.

BPA μg/mL	Nthy‐ori 3‐1		TPC‐1		BCPAP		8505C	
	24 h	48 h	24 h	48 h	24 h	48 h	24 h	48 h
1	37%	22%	8%	1%	3%	2%	5%	3%
4	49%	25%	9%	2%	1%	0.67%	1%	1%
12.5	45%	20%	21%	4%	0.67%	1%	1%	1%
40	35%	13%	6%	3%	1%	1%	1%	1%
100	17%	9%	4%	3%	3%	2%	2%	1%

Exposure to SML (1 μg/mL) reduced Nthy‐ori 3‐1 metabolic activity to 37% of control levels at 24 h, with a further decline to 22% by 48 h. The cytotoxic effect was more pronounced in neoplastic cell lines. TPC‐1, BCPAP, and 8505C cells exhibited profound metabolic suppression at every concentration tested (1–100 μg/mL), with viability frequently falling below 10% at 24 h and near or below 5% at 48 h (Table 2).

Statistical analysis of the metabolic activity data using repeated‐measures ANOVA identified a significant main effect of cell type. Significant interaction effects were also observed for time × concentration and the three‐way interaction of time × concentration × cell type (Tables S2 and S3). Post hoc comparisons indicated that the overall metabolic response of the BCPAP and 8505C cell lines was statistically distinct from that of the Nthy‐ori 3‐1 line (Figure 5).

**FIGURE 5:**
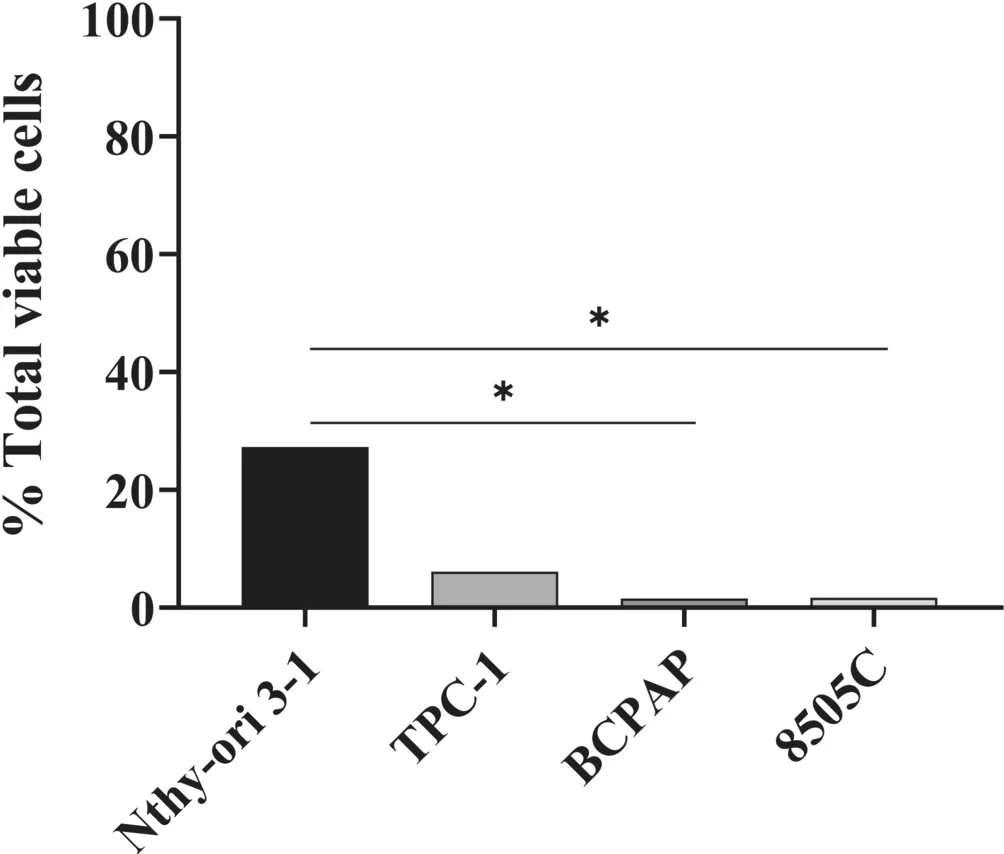
The viability of the four thyroid cell lines exposed to BPA is presented as mean percentages. The comparison included five BPA concentrations and exposure times (24 and 48 h). Repeated measures ANOVA: Results of cell type interactions (Tukey) using the CCK8 assay. * Significant effect of lineage type (Nthy‐ori 3‐1 vs. BCPAP *p* = 0.0148 and Nthy‐ori 3‐1 vs. 8505C *p* = 0.0162). No significant effect was observed between (Nthy‐ori 3‐1 vs. TPC‐1 *p* = 0.1024; TPC‐1 vs. BCPAP *p* = 0.5353; TPC‐1 vs. 8505C *p* = 0.5727 and BCPAP vs. 8505C *p* = 0.9999).

### 3.4 Genotoxic Effects of BPA

Exposure to BPA induced measurable DNA damage in all thyroid cell lines, as quantified by the DNA damage index in the comet assay (Table S4) revealed a genotoxic effect that was generally more pronounced after 48 h of exposure than after 24 h for most concentrations. Analysis of total DNA damage (Classes 1–4) across all tested doses indicated that the BCPAP and TPC‐1 cell lines exhibited greater resistance, whereas the 8505C and Nthy‐ori 3‐1 lines were more susceptible.

At the environmentally relevant dose of 1 μg/mL (LME), the genotoxic impact varied according to cell type (Figure 6). The 8505C anaplastic carcinoma cell line was the most affected, followed by Nthy‐ori 3‐1 and TPC‐1. These three lines displayed a significant frequency of intermediate‐to‐high damage (Classes 2 and 3). In contrast, DNA damage in BCPAP cells at this concentration was predominantly of lower‐magnitude class 1.

**FIGURE 6:**
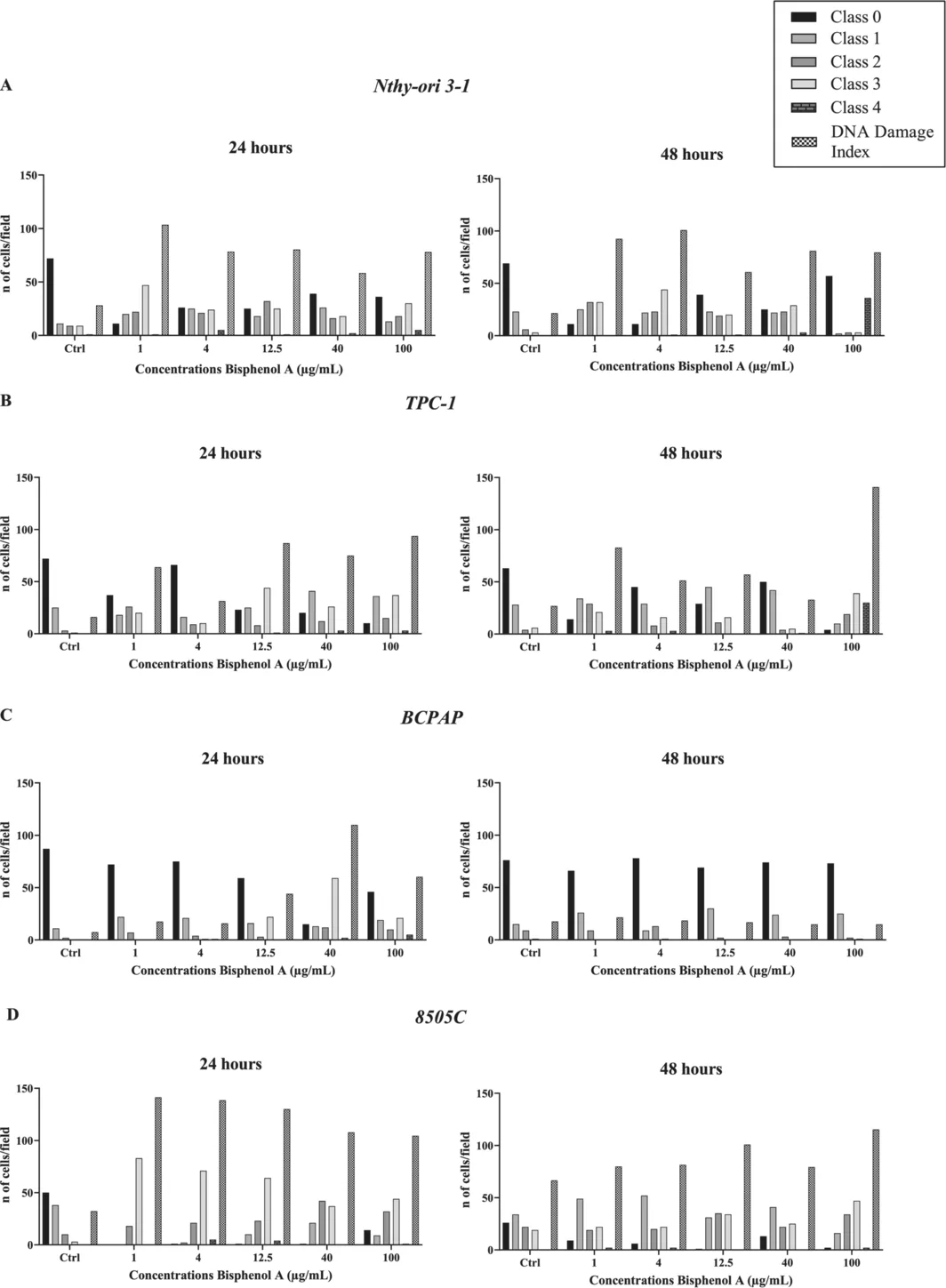
Genotoxic effects of different concentrations of BPA on thyroid cell lines exposed for 24 and 48 h were measured using damage Classes 0–4 and the DNA damage index. Results are presented as mean ± SE.

Exposure to a high concentration of BPA (100 μg/mL) caused substantial DNA damage in all cell lines at both time points. However, the temporal damage patterns differed significantly between the lines. BCPAP cells showed a notable decrease in genotoxicity between 24 and 48 h (*p* < 0.0001), suggesting active DNA repair or the selective loss of heavily damaged cells. Conversely, Nthy‐ori 3‐1 cells displayed a significant increase in damage over the same period (*p* < 0.0001), indicating progressive or irreparable DNA injury (Table S5).

Comparative analysis of the total DNA damage index highlighted the anaplastic 8505C cell line as the most sensitive to BPA‐induced genotoxicity (Figure 7). This line also demonstrated a high baseline level of DNA damage (primarily Classes 2 and 3), even in the absence of BPA exposure (Figure 6D). Among the neoplastic cell lines, BCPAP cells consistently showed the greatest resistance. Statistical comparisons confirmed that the DNA damage index in 8505C cells was significantly higher than that in TPC‐1 and BCPAP cells at 24 h and that BCPAP had a significantly lower damage index than Nthy‐ori 3‐1, TPC‐1, and 8505C at 48 h (Figure 7A,B).

**FIGURE 7:**
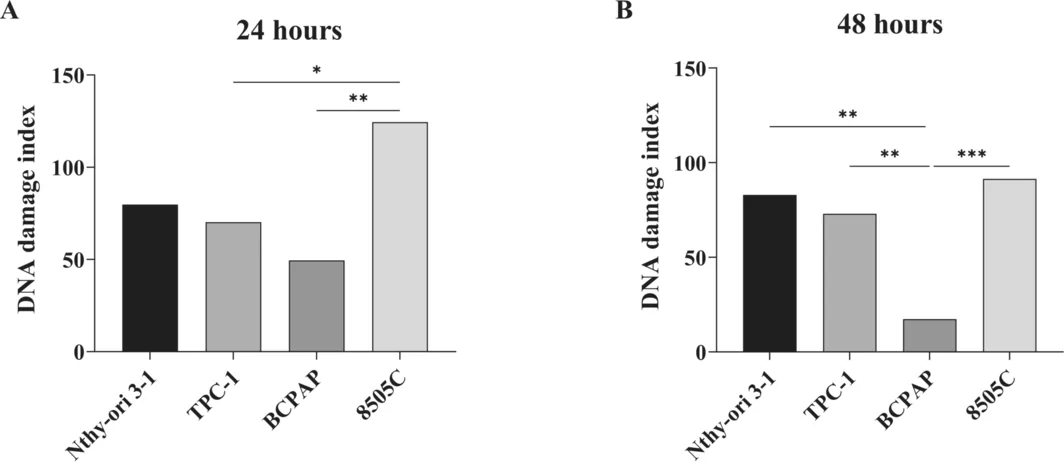
Comparison of DNA damage indices among the four thyroid cell lines. The comparison included the five BPA concentrations, excluding the controls (negative and positive) at 24‐ and 48‐h exposure times. (A) The 8505C line showed significantly higher levels of damage than the BCPAP and TPC‐1 lines (** *p* = 0.0015 and *p = 0.0195, respectively). (B) BCPAP was the most resistant after 48 h of exposure, with significantly lower damage indices than the Nthy‐ori 3‐1, TPC‐1 lines, especially with 8505C (** *p* = 0.0024, ** *p* = 0.0093, and *** *p* = 0.0008). Statistical analysis was conducted using a one‐way ANOVA.

Statistical modeling using GEEs confirmed significant main effects of cell type, exposure time, and BPA concentration on the DNA damage class. Furthermore, all two‐and three‐way interactions (time × concentration × cell type) were statistically significant, underscoring the complex and context‐dependent nature of BPA's genotoxic action of BPA (Tables S5–S7).

## 4 Discussion

This study demonstrates that BPA exerts significant cytotoxic and genotoxic effects on thyroid cells at concentrations ranging from environmentally relevant to supraphysiological levels, including the SML of 1 μg/mL. A key finding was the cell‐context‐dependent nature of these effects, revealing disparate vulnerabilities among normal and neoplastic thyroid lineages.

The normal follicular Nthy‐ori 3‐1 cell line displayed a distinct non‐monotonic dose–response in membrane integrity, with acute sensitivity at low doses, including the SML, followed by a notable recovery at 48 h. This biphasic response suggests the activation of adaptive cellular defense or repair mechanisms following the initial insult, a phenomenon that is less evident in cancer cell lines. In stark contrast, the anaplastic carcinoma line 8505C presented a paradoxical phenotype: marked resistance to membrane lysis at very high BPA concentrations (up to 200 μg/mL) co‐occurred with profound suppression of metabolic activity and extreme sensitivity to DNA damage. This dissociation between membrane integrity and metabolic/genotoxic endpoints underscores that BPA's mechanisms of BPA toxicity extend beyond simple cytolysis.

The pronounced genotoxic and metabolic susceptibility of the 8505C cell line is likely rooted in its distinct molecular pathology. Anaplastic thyroid carcinomas, such as 8505C cells, are characterized by aggressive proliferation, TP53 mutations, and profound genomic instability (Meireles et al. 2007). The TP53 tumor suppressor is a master regulator of cellular stress response, DNA repair, and metabolic homeostasis. Its mutation impairs these critical pathways, rendering cells vulnerable to agents that challenge the genomic or redox balance (Ju et al. 2020; Liberti and Locasale 2016). The severely diminished metabolic activity observed via the CCK‐8 assay aligns with the disruption of core energetic processes, possibly linked to an overburdened or inefficient NADPH‐mediated redox system, which is crucial for both proliferation and antioxidant defense (Oliveira et al. 2021).

Our data support a model in which BPA exposure exploits these pre‐existing vulnerabilities. This compound likely induces DNA damage directly or indirectly via oxidative stress. In 8505C cells, defective TP53‐mediated repair pathways would allow this damage to accumulate, while concomitant disruption of metabolic flux, as evidenced by the CCK‐8 results, further compromises the cellular capacity to mitigate oxidative stress and repair DNA (Moretton and Loizou 2020). This creates a synergistic cycle of metabolic dysfunction and genomic injury, leading to high DNA damage indices. The relative resistance of the BCPAP line, particularly at 48 h, suggests more efficient DNA repair or detoxification mechanisms, potentially related to its different (BRAF V600E) mutational background.

In conclusion, our findings reveal a complex landscape of BPA toxicity in thyroid cells, where the outcomes are critically dependent on cellular origin and genetic background. The normal Nthy‐ori 3‐1 line shows acute low‐dose sensitivity but an adaptive capacity, whereas the neoplastic 8505C line exhibits a unique vulnerability to DNA damage and metabolic disruption despite resisting acute membrane rupture. The demonstration of significant genotoxicity at the regulatory SML is particularly concerning. These results suggest that the current safety thresholds, based primarily on traditional toxicity endpoints, may not sufficiently protect against the more subtle genotoxic and metabolic‐disrupting effects of BPA. This is especially relevant for individuals with pre‐existing thyroid pathologies or genetic susceptibilities, in whom compromised cellular defense mechanisms could amplify the risk. Further investigation into the specific molecular interactions between BPA and dysfunctional pathways in thyroid cancer is warranted to better understand its potential role in carcinogenesis and cancer progression.

## Funding

We are grateful for the São Paulo Research Foundation (FAPESP), Grant number 2020/02167‐3.

## Conflicts of Interest

The authors declare no conflicts of interest.

## Supporting information


**Table S1:** ANOVA results for repeated measures compared by Trypan blue assay (% viability cells*) between cell types, concentrations and times (*n* = 3).
**Table S2:** ANOVA results for repeated measures compared by CCK‐8 assay (% viability cells) between cell types, concentrations and times (n = 3).
**Table S3:** ANOVA for repeated measures for each cell type—without the control group.
**Table S4:** Average of DNA damage classes generated by BPA in 24 and 48 h of exposure.SE Percentage of DNA damage by comet assay. Data are reported as mean ± standard error of tail DNA from 200 cells analyzed for each sample (controls and concentrations).
**Table S5:** Results of the analysis for repeated measures for the full model—GEE—Controls excluded (negative and positive) and damage class = 0.
**Table S6:** Analysis for repeated measures for each cell type—GEE—Excluded controls (negative and positive) and damage class = 0.
**Table S7:** Repeated measures analysis (GEE) between cell type and time—For each type of concentration—Excluded controls (negative and positive) and damage class = 0. Comparison between times (24 h x 48 h) for each cell.

## Data Availability

The data that support the findings of this study are openly available in Repositório de Dados de Pesquisa da Unicamp at https://doi.org/10.25824/redu/OKXGV9.
